# Orai and TRPC channel characterization in Fc*ε*RI‐mediated calcium signaling and mediator secretion in human mast cells

**DOI:** 10.14814/phy2.13166

**Published:** 2017-03-14

**Authors:** Hannah E. Wajdner, Jasmine Farrington, Claire Barnard, Peter T. Peachell, Christine G. Schnackenberg, Joseph P. Marino, Xiaoping Xu, Karen Affleck, Malcolm Begg, Elizabeth P. Seward

**Affiliations:** ^1^ Department of Biomedical ScienceUniversity of SheffieldWestern BankSheffieldUK; ^2^ Academic Unit of Respiratory MedicineUniversity of SheffieldThe Royal Hallamshire HospitalSheffieldUK; ^3^ Metabolic Pathways and Cardiovascular UnitGlaxoSmithKlineKing of PrussiaPennsylvania; ^4^ Respiratory Therapy Area UnitGlaxoSmithKlineStevenageUK

**Keywords:** Calcium, cytokine, Fc*ε*RI, human, mast cell, Orai, STIM, TRPC

## Abstract

Inappropriate activation of mast cells via the Fc*ε* RI receptor leads to the release of inflammatory mediators and symptoms of allergic disease. Calcium influx is a critical regulator of mast cell signaling and is required for exocytosis of preformed mediators and for synthesis of eicosanoids, cytokines and chemokines. Studies in rodent and human mast cells have identified Orai calcium channels as key contributors to Fc*ε* RI‐initiated mediator release. However, until now the role of TRPC calcium channels in Fc*ε* RI‐mediated human mast cell signaling has not been published. Here, we show evidence for the expression of Orai 1,2, and 3 and TRPC1 and 6 in primary human lung mast cells and the LAD2 human mast cell line but, we only find evidence of functional contribution of Orai and not TRPC channels to Fc*ε* RI‐mediated calcium entry. Calcium imaging experiments, utilizing an Orai selective antagonist (Synta66) showed the contribution of Orai to Fc*ε* RI‐mediated signaling in human mast cells. Although, the use of a TRPC3/6 selective antagonist and agonist (GSK‐3503A and GSK‐2934A, respectively) did not reveal evidence for TRPC6 contribution to Fc*ε* RI‐mediated calcium signaling in human mast cells. Similarly, inactivation of STIM1‐regulated TRPC1 in human mast cells (as tested by transfecting cells with STIM1‐KK ^684‐685^ EE ‐ TRPC1 gating mutant) failed to alter Fc*ε* RI‐mediated calcium signaling in LAD2 human mast cells. Mediator release assays confirm that Fc*ε* RI‐mediated calcium influx through Orai is necessary for histamine and TNF *α* release but is differentially involved in the generation of cytokines and eicosanoids.

## Introduction

Mast cells are well known for their contribution to symptoms of allergic disease such as asthma (Metcalfe et al. 1997). Allergic activation of mast cells occurs following cross‐linking of the high‐affinity IgE receptor (Fc*ε*RI) by antigen‐IgE complexes. Fc*ε*RI cross‐linking activates a signaling cascade leading to generation of diacylglycerol (DAG) and inositol triphosphate (IP_3_), which causes calcium release from the endoplasmic reticulum (ER), then calcium influx through calcium permeable channels in the plasma membrane. Both the temporal and spatial properties of the resulting calcium signal are key to controlling exocytosis of preformed mediators such as histamine and de novo synthesis of eicosanoids and cytokines (Di Capite and Parekh 2009). Controlling the release of proinflammatory mediators has long been viewed as an important target for the treatment of diseases in which mast cell activation plays a key role.

Following Fc*ε*RI activation, the production of both DAG and IP_3_ enable calcium entry through both noncalcium selective canonical transient receptor channel family (TRPC) channels, and/or the highly calcium selective store‐operated calcium entry channels (Orai/CRACM). TRPC channels can be directly activated by DAG (Hofmann et al. 1999). IP_3_ however acts indirectly; initiating internal calcium store depletion, which is sensed by the ER resident protein STIM1 (Liou et al. 2005; Yuan et al. 2009), that in turn causes activation of Orai channels (Vig et al. 2006; Liao et al. 2007; Prakriya 2009). Various reports in the literature show TRPC channels can also be activated indirectly, following internal calcium store depletion (Liao et al. 2007; Worley et al. 2007).

The importance of Orai/STIM and TRPC channels for Fc*ε*RI‐mediated mast cell functions in *rodent*s is well described. Studies in mouse fetal liver‐derived mast cells demonstrate that Orai1 or STIM1 knockout impairs Fc*ε*RI‐driven calcium influx and mediator release (Baba et al. 2008; Vig et al. 2008). Further work shows knockdown of Orai1, STIM1 or TRPC5 significantly impairs Fc*ε*RI‐ stimulated calcium influx and degranulation in a rat basophilic leukemia mast cell line (RBL‐2H3 cells) (Ma et al. 2008). It has additionally been demonstrated that TRPC1 and TRPC3 channels contribute to Fc*ε*RI‐mediated calcium signals in RBL‐2H3 mast cells (Cohen et al. 2009). Furthermore, in mouse bone marrow‐derived mast cells (BMMCs), RNAi silencing of TRPC1 significantly inhibits calcium influx and degranulation in response to antigens (Suzuki et al. 2010). Conflicting results were more recently shown in a study, using a TRPC1‐/‐ mouse – that suggested TRPC1 negatively regulated TNF*α* production (Medic et al. 2013); however, a role for TRPC1 in Fc*ε*RI‐mediated mast cell signaling was found nonetheless. Finally, inhibition of Orai channels in primary rat tracheal mast cells prevented allergen‐driven contractions (Rice et al. 2013).

One study in human mast cells (Ashmole et al. 2012) has identified functional Orai/I_CRAC_ currents and calcium signals following Fc*ε*RI activation. Through the use of an Orai selective antagonist, Synta66, they demonstrated that inhibition of Orai channels caused a reduction in the release of proinflammatory mediators. Whether TRPC channels also contribute to Fc*ε*RI calcium signaling in human mast cells is unknown.

In other cell types, there are numerous studies indicating interaction between TRPC1 and Orai channels (Liao et al. 2007, 2009; Lee et al. 2010; Hong et al. 2011; Chen et al. 2014) suggesting dependence of TRPC1 on Orai for its activation (Cheng et al. 2011). Similarly, although TRPC3/6 were classically defined as DAG‐activated channels, the mode of activation of TRPC3/6 are now thought to be more complex. Work has shown that TRPC3/6 channels can be activated via store depletion when an interaction with Orai occurs (Hofmann et al. 1999; Estacion et al. 2004; Liao et al. 2007; Chen et al. 2014). Taken together, data in the literature show that it is still unclear whether TRPC channels contribute to calcium signaling in human mast cells and if there is Orai/TRPC interaction in human mast cells.

In this study, we aimed to evaluate the contribution of Orai channels and TRPC channels to Fc*ε*RI‐mediated mast cell calcium signaling and secretion. We confirmed that in human lung mast cells (HLMCs), Orai conducts calcium influx and is involved in regulating Fc*ε*RI release of both preformed and some, but not all newly synthesized mediators. Conversely, we found no contribution of TRPC channels to Fc*ε*RI‐ mediated calcium signaling in HLMCs or LAD 2 cells, a much used human mast cell line which is widely used as a model for mature mast cells (Kirshenbaum et al. 2003). This study provides evidence that Orai but not TRPC could represent a target for the control of mast cell‐mediated allergic disease.

## Methods

### Ethical approval

The provision of lung tissue and the use of the tissue in this study were approved by the National Research Ethics Service (REC reference: 10/H1010/50). All human subjects gave written informed consent for the use of their tissue.

### Cell culture

All cell types described below were incubated at 37°C in a 5% CO_2_ humidified atmosphere, cells were grown in tissue culture‐treated flasks.

LAD2 were cultured in StemPro‐34 media supplemented with StemPro‐34 nutrient supplement and 2 mmol/L L‐glutamine (all Gibco Life Technologies) in addition to 100 ng/mL recombinant human stem cell factor (rhSCF) (R&D systems). Cells were passaged weekly; media was added to maintain a density of 400,000–500,000 cells/mL.

For data presented in Figure 5, human embryonic kidney cells stably expressing human TRPC6 (HEK‐ TRPC6) were cultured in DMEM containing 10% FCS and 400 *μ*g/mL geneticin (Gibco) to select for TRPC6 expression.

For data presented in Figure S5, human and rat TRPC3 and TRPC6 channels were heterologously expressed in human embryonic kidney 293 (HEK293) cells, using BacMam transduction. All human and rat TRPC3 and TRPC6 BacMam reagents were generated at GlaxoSmithKline (King of Prussia, PA). HEK293 cells were grown in 6‐well culture dishes, using DMEM F‐12 medium supplemented with 10% FBS and 1% Pen/Strep. 6–12% TRPC3 or TRPC6 BacMam virus were added to 6‐well culture dishes 24~48 h before experiments. HEK 293 cells were detached from the culture dish using trypsin solution (0.25% trypsin + 0.1% EDTA) and stored in the culture medium at room temperature for patch‐clamp experiments within 5 h.

### Human lung mast cell culture

Nonlesional tissue from lung resections was obtained after surgery. Tissue was enzymatically digested by methods adapted from (Sanmugalingam et al. 2000; Cruse et al. 2005) and mast cells were isolated, using the Dynal^®^ magnetic bead system using CD117 antibody‐coated beads (Miltenyi Biotech), as described by Okayama et al. (1994). Human Lung Mast Cell (HLMCs) were cultured in DMEM+Glutamax media (Gibco) containing 1% antibiotic–antimycotic solution (Sigma), 1% non‐essential amino acids, 10% fetal calf serum (Gibco) and supplemented with 100 ng/mL human stem cell factor, 50 ng/mL IL‐6 and 10 ng/mL IL‐10 (R&D systems). For histamine assays mast cells were isolated from human lung tissue by a method described by Lewis et al. (2013) and used within 24 h.

### Quantitative PCR

RNA was extracted from 250,000 cells/donor (RNeasy Kit. Qiagen), the optional DNase clean up step was performed. RNA concentration/purity was determined, using a nanodrop (Thermo‐scientific), and cDNA conversion was completed, using a high capacity RNA to cDNA kit (Applied Biosystems) according to the manufacturer's instructions. QPCR was run on a BioRad thermocycler machine, using 5 *μ*g of cDNA per reaction. Primers were custom designed and optimized by PrimerDesign©, ‘Precision mastermix for the Bio‐Rad iCycler with SYBR green’ was used throughout. Reverse transcriptase and non‐template controls were used to verify that there was no genomic DNA contamination and melt curves were analyzed to assess the primer specificity. The geNorm analysis was performed to determine the most consistently expressed housekeeping gene in the samples of interest. Raw threshold cycle (Ct) values were then normalized to the housekeeping gene (18sRNA) and data are expressed as 2^∆Ct.

### Microarray

Microarray experiments were performed, using an Agilent SurePrint G3 Gene Expression 8 × 60K one‐color microarray system, which enables estimation of absolute levels of gene expression between arrays. RNA was collected from 500,000 LAD2 or HLMC cells, using RNeasy Kit (Qiagen) according to manufacturer's instructions. The data were normalized to the 75th percentile intensity of all non‐control probes according to Agilent instructions, allowing comparison across arrays.

### Immunocytochemistry

Cells were fixed with 4% paraformaldehyde + 4% sucrose (pH7.4) for 10 min followed by washing in PBS and permeabilization with PBS + 0.1% Triton‐X‐100 (Sigma) for 15 min. Blocking solution of 0.02% Triton‐X‐100 and 0.2% fish skin gelatin (Sigma) was applied for at least 2 h at room temperature before addition of primary antibody overnight at 4°C (TRPC1, Alomone ACC‐010; TRPC6, Origene TA306349; Rabbit polyclonal IgG, Abcam ab27472 each used at 1:200). Secondary antibodies at concentrations of 1:1000 (Anti‐Rabbit Alexa Fluor 488, polyclonal, Invitrogen) were incubated for 1.5 h at room temperature before the coverslips were mounted onto glass slides, using DAPI‐Fluoromount G (Southern Biotech). Images were taken with an Olympus FV1000 confocal microscope and quantification was performed on Image J. Image quantification is represented as mean fluorescent intensity minus background.

### LAD2 cell transfection and preparation of STIM1 constructs

A Neon^®^ Life Technologies electroporation system was used. 100,000 cells were used per transfection condition with pulse duration of 30 msec at 1600 mV. Cells were used in experiments 48 h after transfection. Human STIM1‐WT‐YFP/MO91 with a CMV promoter was bought from Addgene. STIM1 (KK684‐685EE)/pcDNA3.1 with a CMV promoter was kindly donated by Ambudkar lab. Constructs were grown up in DH5*α* competent cells (Sigma) and then extracted, using a GenElute^™^ Plasmid midiprep kit (Sigma) as per manufacturer's instructions. DNA was concentrated to 1 *μ*g/mL and purified DNA was sequenced before use to confirm sequence integrity.

### Calcium imaging

Cells were loaded with fura‐2 AM (1 *μ*mol/L) (Invitrogen Molecular Probes) in HLMC culture media (omitting antibiotic‐antimycotic) for 30 min at 37°C. An inverted microscope (Axiovert S100 TV, Zeiss, Cambridge, UK) equipped with a 40x oil immersion objective (NA 1.3, Zeiss) was used. Cells were alternately illuminated at 340 and 380 nm with a 20 msec exposure time. (Polychrome IV, TILL Photonics, Munich, Germany). Emitted light was passed through a 510 nm band pass filter and collected by a 512B Cascade CCD camera (Photometrics, Tucson, AZ) and images were acquired at 0.5 Hz. Recording chamber was continually superfused with external solution (in mmol/L; 120 NaCl, 10 KCl, 10 HEPES, 2 MgCl_2_, 2 CaCl_2_, 10 Glucose (300 mOsm/L, pH 7.3, NaOH)) at a rate of approximately 1.5 mL/min. MetaMorph^®^ Meta imaging software (Molecular Devices, Sunnyvale, CA) was used to analyze all calcium imaging experiments and background signal was subtracted.

### Histamine assays

Inhibitory compounds were added to cells 5 min (time of onset in patch‐clamp experiments performed in this lab showed that 5 min was sufficient time for Syntaa66 to inhibit Orai currents) prior to stimulation with anti‐Ig*E,* (the previously established EC_80_ concentration) (Sigma) for 25 min at 37°C in a 5% CO_2_ humidified incubator. Samples were diluted in PBS and spun at 1500 RPM for 10 min, supernatants were then collected for histamine analysis. Histamine levels were determined as a percentage of total histamine, where total values were obtained from equivalent cells lysed with 0.5% perchloric acid. Spontaneous release was measured from supernatants without addition of anti‐IgE. Histamine levels were determined, using a fluorimetric method first described by Siraganian (1975) and later modified by Ennis (1991).

### Lipid & Cytokine mediator release assays

Eicosanoid and cytokine/chemokine concentrations were determined from supernatants of isolated primary HLMCs 7–10 days post‐purification. Cells were initially pre‐sensitized with 300 ng/mL human IgE (Calbiochem) for 24 h before a 25 min/24 h stimulation with anti‐IgE (Sigma) at 37°C for eicosanoid/cytokine mediator release, respectively. Inhibitors or vehicle controls were pre‐incubated for 5 min prior to addition of anti‐IgE. Supernatants were removed and stored at −80°C until assays were performed.

Prostaglandin D_2_ content was measured, using a Prostaglandin D_2_‐MOX EIA kit, TNF*α* concentration was determined, using a QuantiGlo^®^ Chemiluminescent ELISA (R&D Systems) and cytokine/chemokines, using the Proteome Profiler^™^Array ‐ Human cytokine panel array A (R&D systems Abingdon, UK) each in accordance with the manufacturer's instructions. Plates were read, using a FLUOstar OPTIMA luminometer (BMG LABTECH), using OPTIMA software; 0.5 sec/well read time.

### Electrophysiology

Whole cell patch clamp experiments were conducted at room temperature (~22°C). Cells were placed in a small chamber and continuously perfused with an external solution (~3 mL/min). Electrodes were made from glass capillary tubes and had a resistance of 3–4 MΩ when filled with internal solutions (for TRPC3 current in mmol/L: 140 CsCl, 5 Na_4_EGTA, 10 HEPES; pH=7.2; for TRPC6 current in mmol/L: 130 CsCl, 5 EGTA, 5.5 MgCl_2_, 5 Na_2_ATP, 0.1 Na‐GTP, 5 HEPES; pH=7.2). AXOPATCH 200B amplifier and pCLAMP software (version 8, Molecular Devices) were used for data acquisition. Seal between the cell membrane and electrode was made in an external solution containing (mmol/L) 140 NaCl, 4 KCl, 1 MgCl_2_, 0.2 CaCl_2_, 10 Glucose, 10 HEPES; pH=7.4. Cell membrane capacitance was canceled electronically and the series resistance was compensated by about 70%. External solution was then switched to the one omitting CaCl_2_ but with 2 mmol/L Na_4_EGTA (same other components) in order to minimize desensitization of TRPC3 and TRPC6 current. TRPC3 or TRPC6 current was activated, using agonist GSK1702934A applied to the bath solution. To record TRPC3 or TRPC6 current, a ramp voltage protocol was applied every 10 sec for as long as the experiment lasted. The ramp protocol stepped from a holding potential of −60 mV to −80 mV for 40 msec and then depolarized to +80 mV in 400 msec, finally stepped back to −60 mV after having spent 40 msec at +80 mV. TRPC3 or TRPC6 current gradually increased as the cell was perfused with GSK1702934A. The TRPC3 or TRPC6 current was measured as the average current at −80 or +80 mV. The time course of current was plotted for the whole experiment.

### Patch clamp data analysis

The effect of agonist GSK1702934A was calculated as %Current activation = 100×(I_D_‐ I_C_)/(I_max_‐ I_C_), where I_D_ was the current amplitude measured at the peak response of a particular concentration of GSK1702934A, I_C_ was the control current amplitude measured before GSK1702934A application, and I_max_ was the current amplitude at the maximal response (1 *μ*mol/L for TRPC3 and 3 *μ*mol/L for TRPC6). The averaged data were fit, using 4‐Parameter Logistic Equation (Origin 7.0 software) to calculate half maximal activation concentration (EC_50_). Data were expressed as mean ± SE (N). Prism 5 software was used for comparing the mean of EC_50_, and *P* < 0.05 was considered statistically significant.

### Statistical analysis

Paired/unpaired students t‐test and one‐way ANOVA with Bonferonni or Tukey's post hoc tests were used as appropriate and performed, using GraphPad Prism. A *P*‐value of *P* < 0.05 was considered to indicate statistical significance. Data expressed as mean ± S.E.M.

## Results

### TRPC1 and 6 are expressed in LAD2 and HLMCs

**Figure 1:**
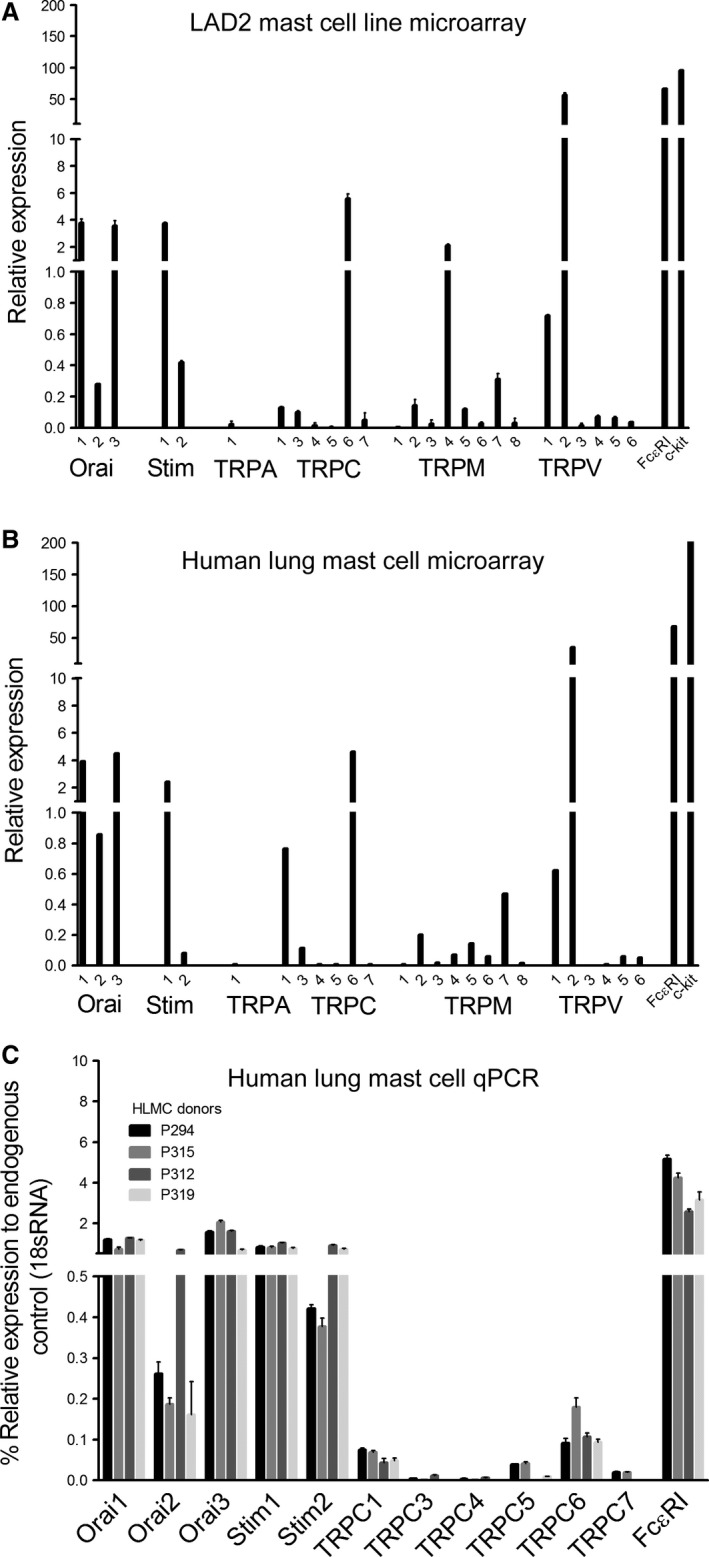
*Orai and TRPC subtypes are expressed in LAD2 and HLMC cells at mRNA level*. Microarray data was normalized to the 75th percentile of all non‐control probes, according to Agilent instructions. (A) LAD2 mRNA expression from three independent RNA extractions ±SEM (B) HLMC mRNA expression from one HLMC donor. (C) Quantitative PCR to assess the expression of Orai, STIM and TRPC mRNA in HLMCs. Expression was normalized to 18S endogenous control and expressed as % relative to 18S. –RT and NTC controls were performed to show no genomic contamination was present. SYBR green probes were used and melt curves plotted to assess primer specificity.

To get a comprehensive view of the calcium permeable channels which may putatively contribute to FcεRI signaling in human mast cells, and validate the LAD2 human mast cell line as a suitable model for primary HLMCs, we first performed a microarray analysis of mRNA expression in the two cell types. Analysis of the microarray data show expression of Orai1, 2 and 3, with Orai 1 and 3 expressed at levels of 3‐4 compared to Orai 2 at <1, in both cell types **(**Fig. 1). Similarly, mRNA expression of TRPC1, 3 and 6 were detected (at levels of <1, <0.2 and, <6, respectively). In addition to the TRPC channels specifically investigated in this study, the microarray data also provided novel evidence for the expression of TRP channel members from the melastatin TRP family and the vanilloid TRP family, providing useful insight into direct future research in the field. Interestingly, TRPM7 (<0.4) and P2X7 (<2 ‐ data not shown) were detected at similar levels to the TRPC family subtypes. Both these channels have previously been functionally characterized in human mast cells (Wykes et al. 2007; Wareham et al. 2009; Wareham and Seward 2016); therefore, demonstrating that this level of mRNA expression could be sufficient to provide a functional contribution to cell physiology.

**Figure 2:**
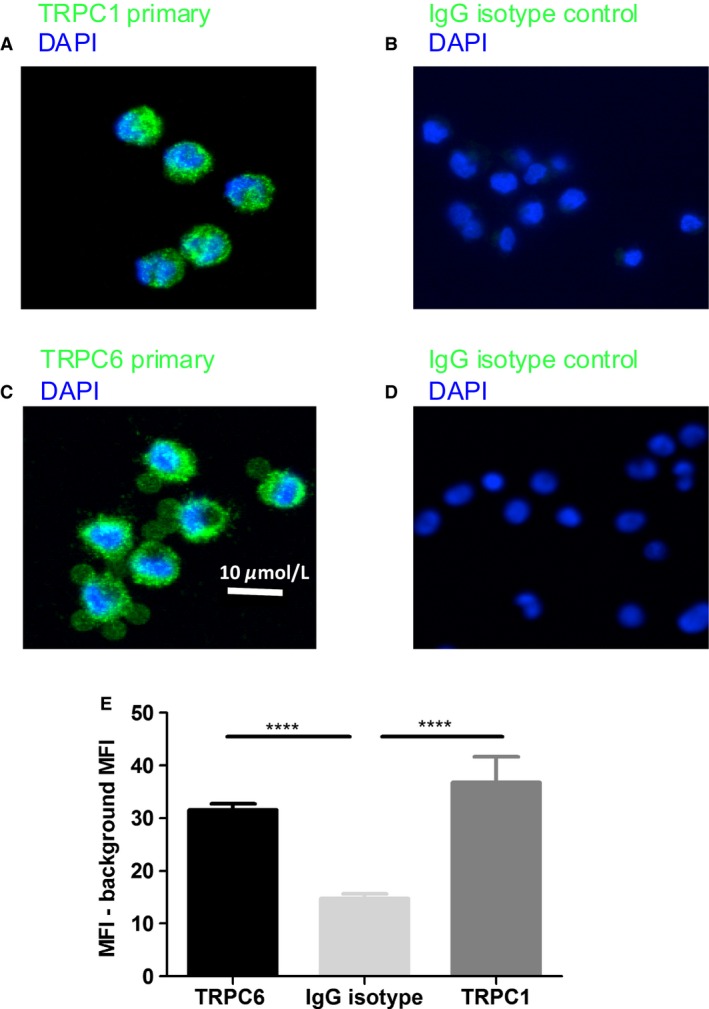
*TRPC1 and 6 are expressed in HLMCs at protein level*. Immunocytochemistry to assess TRPC channels in HLMC cells, (A) anti‐TRPC1 staining, (B) IgG isotype control. (C) anti‐TRPC6 staining (D) IgG isotype control. Bar graph shows quantification of mean intensity of cells in each conditions ± SEM. Results were analyzed, using one‐way ANOVA with Tukey's multiple comparison test. *P < 0.01 ****P < 0.0001.

In order to verify the consistency of expression of TRPC subtypes in HLMCs between different lung donors, qPCR was performed on four additional lung donors. The results (Fig. 1) confirm that TRPC1 and 6 are the only TRPC family members that are expressed in HLMCs, and this was uniform across multiple donors. Based on this evidence, immunocytochemistry experiments were performed to confirm protein expression of TRPC1 and C6. TRPC1 (TRPC1 Ab mean intensity =36.8 ± 4.8 *n* = 31) and TRPC6 staining were observed in HLMCs (TRPC6 Ab mean intensity 31.58 ± 1.1 *n* = 41, IgG isotype control mean intensity =16.7 ± 0.7 *n* = 69 cells) both at levels significantly greater than in the negative controls, (Fig. 2). The staining shows a largely intracellular diffuse pattern with no obvious surface staining. Positive expression of TRPC1 in LAD2 cells was similar (Fig. S1); TRPC1 Ab mean intensity = 142.7 ± 1.12 *n* = 41, IgG isotype control 45.7 ± 1.4 *n* = 35 cells. As a whole, the data presented in Figures 1 & 2 show novel evidence for the expression of TRPC1 and C6 in HLMCs and LAD2 cells, and confirm expression of Orai channels in HLMCs as previously reported (Ashmole et al. 2012).

### Synta66‐sensitive channels (Orai and Orai‐regulated channels) contribute to FcεRI‐mediated calcium entry in HLMCs and LAD2s

**Figure 3:**
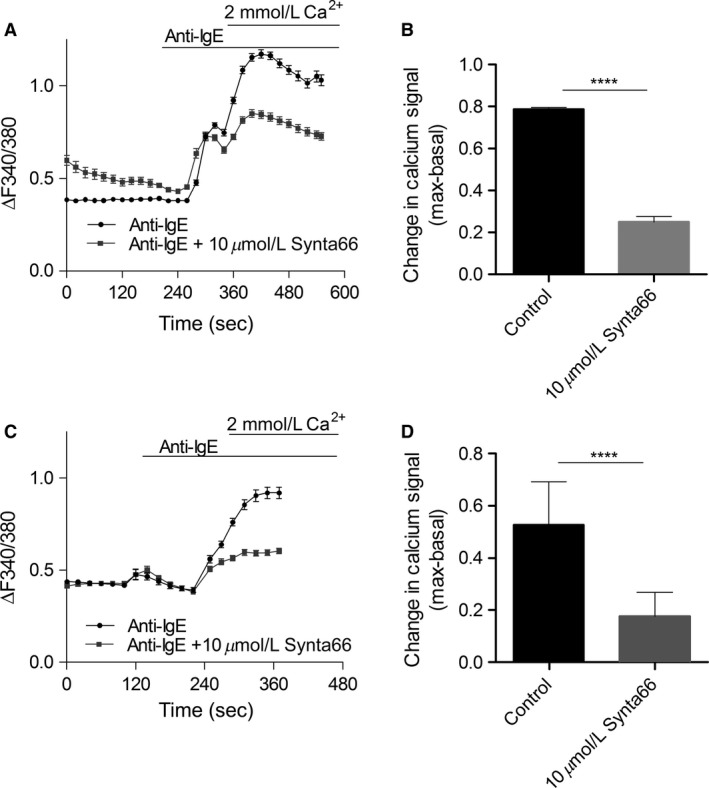
*FcεRI activated calcium influx in HLMCs and LAD2s is inhibited by Orai inhibitor, Synta66*. Calcium imaging of fura 2‐AM loaded HLMCs and LAD2s. HLMCs and LAD2s were incubated overnight with 300 ng/mL IgE (A) Calcium signal over time, 3 *μ*g/mL anti‐IgE and 10 *μ*mol/L Synta66 were bath applied as indicated by the horizontal bars. This is a representative trace from 3 HLMC donors tested (B) Bar graph showing mean fluorescence change in calcium signal (t300sec–t0sec) for conditions ± Synta66. *n* > 40 for each donor over 2–3 experiments. (C and D) Same as A and B but for LAD2 cells. Results were analyzed, using unpaired students t‐test ***P < 0.001, ****P < 0.0001.

To investigate the identity of channels underlying calcium signaling induced by Fc*ε*RI activation, calcium imaging experiments were performed on isolated cells loaded with Fura‐2AM. A calcium ‘add‐back’ protocol was performed where the stimulus was applied in the absence of extracellular calcium to allow separation of the calcium signal induced by ER store release from the calcium influx through plasma membrane channels. As shown in Figure****** 3, the anti‐IgE‐initiated calcium signal exhibited an initial fast increase in signal (store max – basal = 40/60 sec for LAD2/HLMC) followed by a signal rise which was more sustained (max‐basal = 130/100 sec for LAD2/HLMC). Pre‐application of 10 *μ*mol/L Synta66 (to block Orai contribution) inhibited the anti‐IgE induced change in fura‐2 fluorescence by 69% in HLMCs, and by 67% in LAD2 cells. Note that given the nonlinear relationship between the fura‐2 fluorescence ratio (340/380 nm) and Ca^2+^ concentration, the actual decrease in free calcium in the cells is actually predicted to be even greater. Synta66 had no significant effect on the store component of the signal in either cell type. Although interestingly, the store signal is seen to be consistently higher in HLMCs compared to LAD2 cells (~0.3 vs 0.1 ∆signal). In HEK cells over‐expressing Orai1/STIM1, 10 *μ*mol/L Synta66 caused 94 ± 3% inhibition of I_CRAC_ currents (data not shown). Work by Di Sabatino et al. 2009 provides a comprehensive list of targets which have been shown to be insensitive to 10 *μ*mol/L Synta66 treatment giving confidence in the selectivity of this compound at this concentration. Compound structures for Synta66 are shown in Derler et al. 2013. Together with the expression data shown in Figure 1, data in Figure 3 show compelling evidence that there are functional Orai channels and/or Orai‐regulated channels in HLMCs and LAD2 cells.

### STIM1‐regulated TRPC1 does not contribute to FcεRI‐mediated calcium entry in LAD2 cells

**Figure 4:**
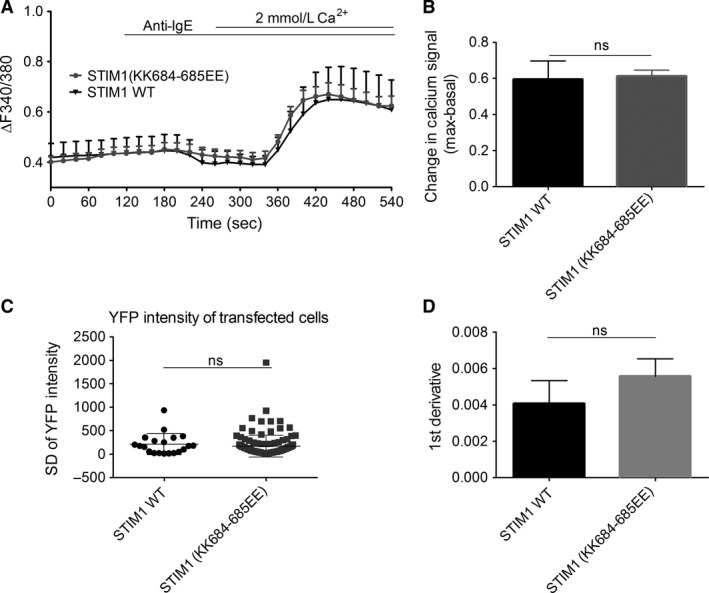
*STIM1 regulated TRPC1 does not contribute to FcεRI‐mediated calcium entry in LAD2 mast cells*. Calcium imaging of Fura 2‐AM loaded LAD2 cells transfected with YFP‐STIM1‐WT or YFP‐STIM1 KK684‐685EE. LAD2s were incubated overnight with 300 ng/mL IgE. (A) Calcium signal over time, 1 *μ*g/mL anti‐IgE applied as indicated by horizontal bars. (B) Bar graph showing mean fluorescence change in calcium signal (max‐basal) (C) Bar graph showing standard deviation of YFP intensity in transfected cells. (D) Bar graph showing the 1st derivative of t = 400s. All data are shown as mean ± SEM. *n* > 10 cells *N* = 3. Only YFP‐expressing cells included for analysis. Results were analyzed, using unpaired students t‐test, ns *P* > 0.05.

In order to specifically assess whether TRPC channels contribute to Fc*ε*RI‐ induced calcium signaling in human mast cells, two routes of investigation were undertaken. Based on the expression data shown in Figure****** 1, the potential functionality of TRPC1 and TRPC6 were focussed on. At present, there are no specific pharmacological tools to inhibit TRPC1; therefore, in order to test whether TRPC1 was functionally active in human mast cells, LAD2 mast cells were transfected with an STIM1‐KK^684‐685^EE mutant which renders STIM1‐regulated TRPC1 inactive (Cheng et al. 2011). The Lys 684‐685 region of STIM1 has been shown to interact electrostatically with TRPC1 aspartate residues to control gating of TRPC1, but not to interact with Orai channels. Thus, mutating this Lys region of STIM1 to glutamate reverses the charge so that STIM1 is no longer able to activate TRPC1 (Zeng et al. 2008). YFP tagged ‐ STIM1‐KK^684‐685^EE or STIM1‐WT constructs were transfected into LAD2 human mast cells in order to monitor the resulting changes in calcium signal. Time lapse imaging experiments of LAD2 cells transfected with the YFP‐tagged constructs are shown in Figure S2; the YFP intensity is significantly increased at the plasma membrane after store‐depletion with thapsigargin (normalized YFP intensity; WT = 1.00 to 1.18 ± 0.06 and STIM1‐KK^684‐685^EE = 1.00 to 1.13 ± 0.07), indicating the STIM1 constructs are able to translocate to the plasma membrane following store depletion and confirming expected functionality of the constructs. When calcium imaging experiments were performed on the transfected LAD2 cells, there was no significant difference following Fc*ε*RI receptor activation with a mean signal change of 0.6 ± 0.1 in both STIM1‐WT and STIM1‐KK^684‐685^EE cells (Fig. 4). To determine that the level of construct expression was not variable between the mutant and control conditions, the YFP intensity of cells transfected with each construct was quantified, and found to not be significantly different. In the STIM1‐WT cells, the S.D. of YFP intensity was 212.0 ± 50 (*n* = 20) and in the STIM1‐KK^684‐685^EE cells, it was 167.1 ± 20.4 (*n* = 30). Thus, unlike store‐operated calcium signals evoked in HSG cells (Cheng et al. 2011) and rodent mast cells (Cohen et al. 2009; Suzuki et al. 2010), our data indicate that STIM1‐regulated TRPC1 does not contribute to Fc*ε*RI‐ induced calcium signaling in LAD2 human mast cells.

### TRPC3/6 channels do not contribute to FcεRI‐ induced calcium entry in HLMCs

**Figure 5:**
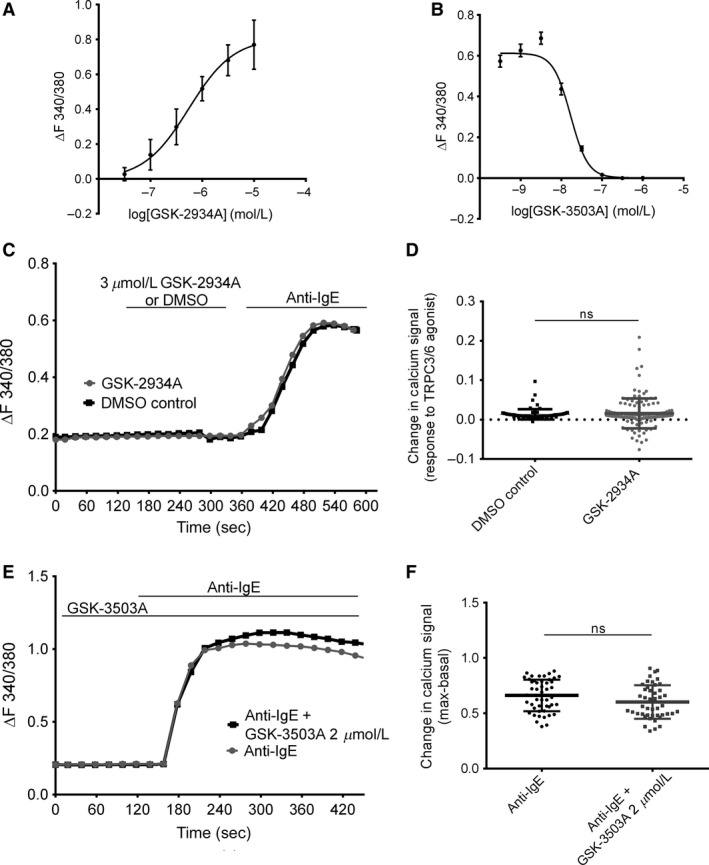
*TRPC3/6 agonist and antagonist have no effect on calcium signaling in human mast cells*. Calcium imaging of Fura 2‐AM loaded HEK‐TRPC6. (A) Concentration‐response curve in HEK‐TRPC6 cells – summarizing change in calcium signal to varying concentrations of GSK‐2934A agonist. (B) Effect of varying concentrations of antagonist GSK‐3503A to 3 *μ*mol/L GSK‐2934A induced calcium signal in HEK‐TRPC6 cells. *n* > 20 cells for each concentration performed over 2‐3 independent experiments. Data shown as mean ± SEM (C‐F) Calcium imaging of fura 2‐AM loaded HLMCs. (C) Calcium signal over time; DMSO vehicle control/3 *μ*mol/L GSK‐2934A was applied as indicated by the horizontal bars, followed by anti‐IgE, 1 *μ*g/mL. (D) Scatter graph showing max‐basal calcium signal of TRPC3/6 agonist (before anti‐IgE application) (E) Calcium signal over time. Effect of GSK‐3503A on anti‐IgE induced calcium entry. Solutions applied as indicated by horizontal bars. 2 *μ*mol/L GSK‐3503A pre‐incubated for 10 min and present in solution throughout the experimental duration. (F) Scatter graph showing max‐basal calcium signal of anti‐IgE response in each cell. Lines in bar graphs show mean ± SEM, calcium signal over time show representative traces. *n* > 45 cells for each condition, *N* = 3 donors. Results were analyzed, using unpaired students t‐test, ns *P* > 0.05.

Recently discovered potent and selective TRPC3/6 agonists and antagonists (Washburn et al. 2013; Seo et al. 2014) were used to investigate the possible contribution of TRPC3/6 to Fc*ε*RI‐induced calcium influx in human mast cells. GSK2833503A (GSK‐3503A) is a selective inhibitor of TRPC3 and TRPC6 with at least 100‐fold selectivity over other calcium‐permeable channels (example 19 in Washburn et al. 2013). GSK1702934A (GSK‐2934A) is a potent TRPC3/6 agonist (Figure S5) and does not stimulate TRPV4, TRPA1, M1, M4, CaV1.2, hERG, NaV1.5, or CXCR5 receptors at concentrations <10 *μ*mol/L. Compound structures for GSK‐2934A and GSK‐3503A are illustrated in Figure****** 5. The activity of the TRPC3/6 compounds to change intracellular calcium concentrations was performed in a HEK cell line over‐expressing TRPC6 (Fig. 5A, B). We show that the agonist GSK‐2934A induces calcium signals in HEK‐TRPC6 cells (pEC_50_=6.6) and the antagonist GSK‐3503A inhibits TRPC3/6‐mediated calcium signals (pIC_50_=7.8). From these data approximate EC_80_ values were used for further investigations (Fig. 5C–F). Further, characterization of the TRPC3/6 agonist, GSK‐2934A was performed in whole cell patch clamp electrophysiology experiments shown in Figure S5. The data presented in this figure support that the GSK‐2934A compound activates TRPC3/6 channels with similar potency to that observed in calcium imaging assays. Here, we also show that GSK‐2934A activates rat TRPC3 and C6 as well as human TRPC3 and C6, no difference in the potency of the compounds was seen between rat and human models. These selective pharmacological tools allow novel investigation into whether TRPC3/6 channels are contributing to mast cell signaling and function.

Calcium imaging in HLMCs activated with 3 *μ*mol/L GSK‐2934A, the TRPC3/6 agonist (Fig. 5C,D) did not induce a significant increase in calcium signal,(0.01 ± 0.001, *n* = 126 cells *N* = 5 donors), however, the same cells did respond to anti‐IgE at typically observed magnitude (Δ0.6), applied at the end of the experiment as an experimental control (Fig. 5C)**.**

Previous studies show TRPC6 channels require signaling proteins to initiate their translocation to the plasma membrane (Cayouette et al. 2004; Monet et al. 2012). Therefore, it is possible that a signal change in response to the TRPC3/6 agonist was not observed in our experiments (Fig. 5C) because TRPC6 channels were not present in the plasma membrane. To explore this, the effect of the TRPC3/6 antagonist (2 *μ*mol/L GSK‐3503A) on Fc*ε*RI‐ induced calcium influx was investigated. Fc*ε*RI activation induced mean max‐basal change of 0.6 ± 0.02 (*n* = 49 *N* = 3 donors) in control HLMC, which was the same as the 0.6 ± 0.02 response seen in GSK‐3503A treated cells (*n* = 45 *N* = 3 donors). These data demonstrate that when using selective pharmacological tools, there is no evidence for a contribution from TRPC3/6 to Fc*ε*RI‐initiated calcium signaling in HLMCs.

Calcium imaging in HLMC and LAD2 mast cells suggest that only Synta66‐sensitive Orai calcium channels are contributors to Fc*ε*RI‐induced calcium signaling, suggesting that the Orai channels are likely to have a critical role in driving Fc*ε*RI‐dependent mast cell functions. To determine whether Orai‐dependent calcium signaling is critical and wholly responsible for mast cell mediator release further experiments were performed, utilizing the Orai selective inhibitor, Synta66. The data shown in Figure S4 confirms the lack of a cytotoxic effect of Synta66 on HLMC cells when incubated over a 24 h time period.

### FcεRI‐activated histamine and TNFα release is significantly inhibited by Synta66 in HLMCs

**Figure 6:**
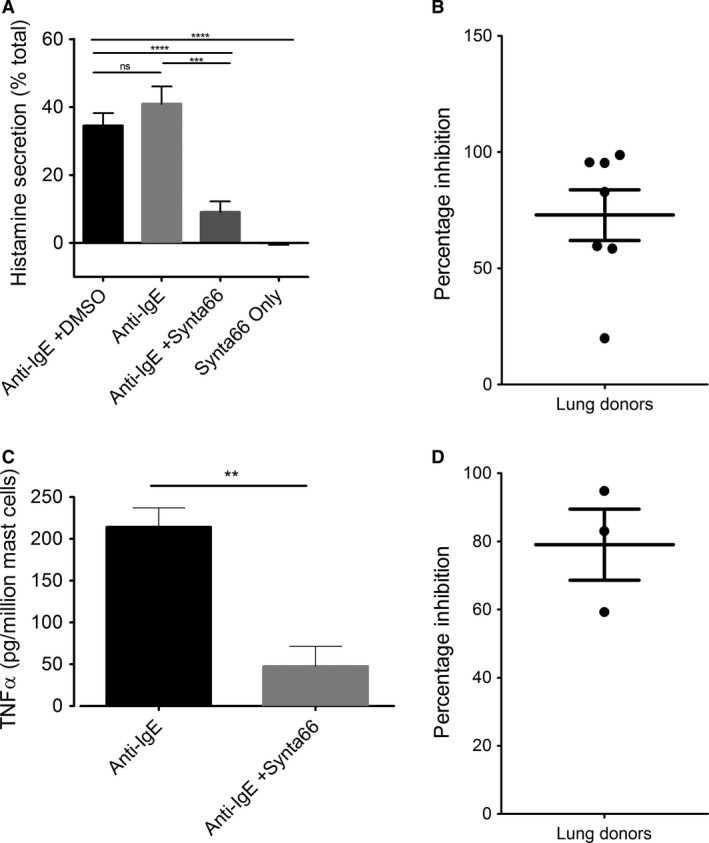
*Synta66 significantly inhibited FcεRI stimulated histamine and TNFα secretion in HLMCs*. Freshly isolated impure HLMCs were stimulated with anti‐IgE and histamine release was measured as a percentage of total histamine present in equivalent cell numbers in each donor. Each data point represents *n* = 40,000 HLMCs from individual lung donors. (A) Histamine release when cells were stimulated with 3 *μ*g/mL anti‐IgE alone (*N* = 7) anti‐IgE in the presence of 0.1% DMSO (*N* = 4), 10 *μ*mol/L Synta66 (*N* = 7), and with 10 *μ*mol/L Synta66 alone (*N* = 5). (B) Percentage inhibition in each donor. (C) TNFα was measured from isolated primary HLMCs cultured for 7 days in enriched media and incubated overnight with 300 ng/mL IgE. Cells were pre‐incubated for 5 min with Synta66 (10 *μ*mol/L) prior to stimulation with 3 *μ*g/mL anti‐IgE. Supernatants were harvested 24 h after challenge with anti‐IgE. (D) Percentage inhibition in each donor (*N* = 3). Bar represents mean ±SEM. Results were analyzed, using one‐way ANOVA with Tukey post test. *= means were significantly different: **P < 0.01,***P < 0.001, ****P < 0.0001.

Histamine is a preformed mediator, released via exocytosis from mast cell granules by a calcium‐dependent process (Douglas and Ueda 1973). A five minute pre‐application of 10 *μ*mol/L Synta66 caused significant inhibition of Fc*ε*RI‐ mediated histamine (73% ±10, *N* = 7 donors) secretion in HLMCs (Fig. 6)***.*** This shows the dependency on Synta66‐sensitive channel‐driven calcium influx, in Fc*ε*RI‐ induced histamine release.

TNF*α* can be both pre‐stored and secreted through the regulated pathway as well as de novo synthesized and secreted (Gordon and Galli 1991). TNF*α* production and secretion was measured from HLMC supernatants collected 24 h after Fc*ε*RI activation, in the presence and absence of Synta66. The compound caused a mean percentage inhibition of 79% ± 10 *N* = 3 donors, demonstrating that Orai‐ mediated calcium entry is also a critical requirement for the secretion of TNF*α* from HLMCs.

### FcεRI‐activated eicosanoid and cytokine release are differentially inhibited by Synta66 in HLMCs

De novo synthesized lipid mediators and cytokines that are also significant contributors to allergic inflammation and activation of other immune system cells and their production is also a calcium‐dependent process (Hogan et al. 2003; Di Capite and Parekh 2009). Therefore, we assessed the contribution of Synta66‐sensitive channels to Fc*ε*RI‐mediated de novo eicosanoid release.

**Figure 7:**
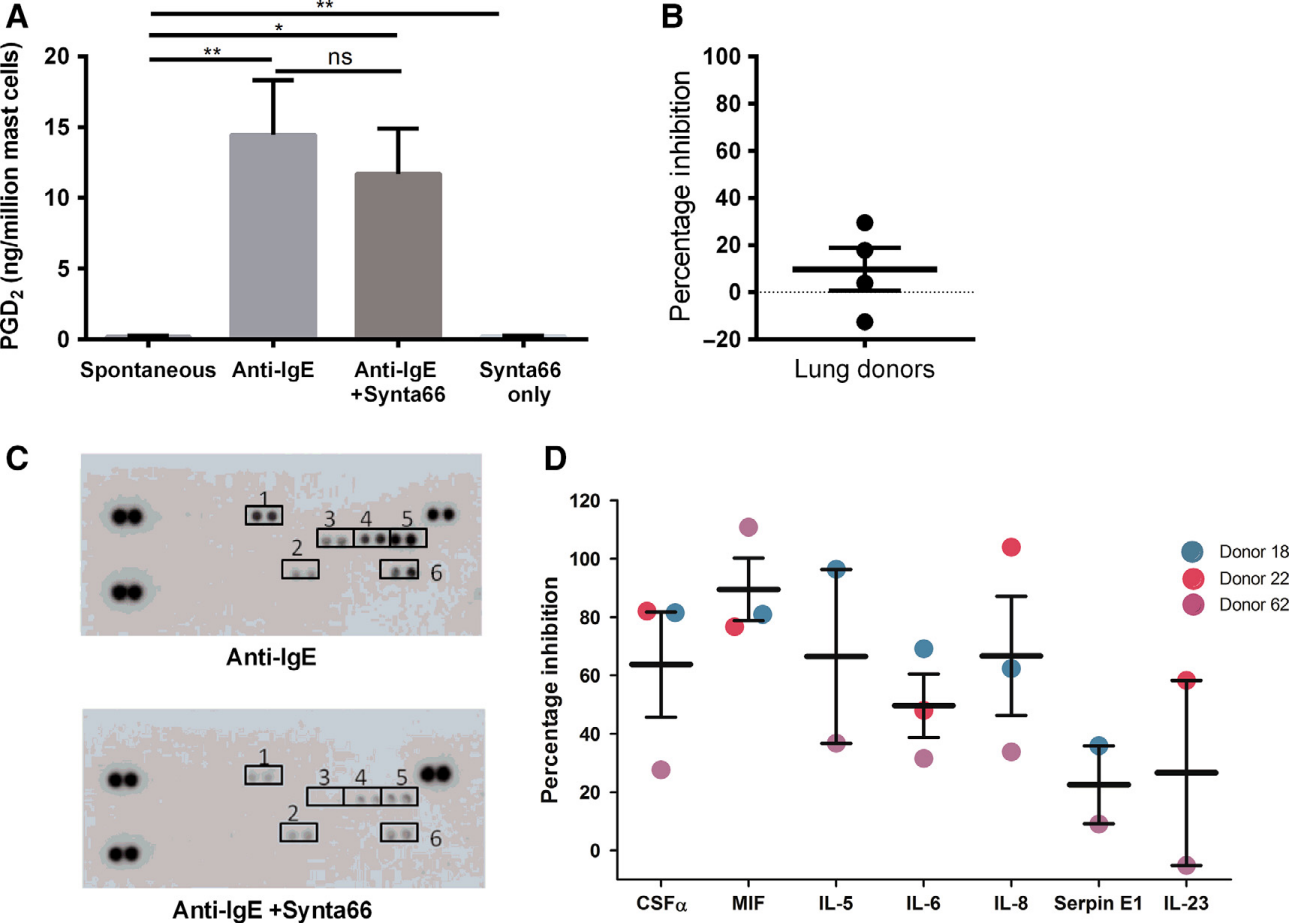
*Synta66 had differential effects on FcεRI stimulated Prostaglandin D2 and cytokine release in HLMCs*. Prostaglandin D2 and cytokine release was measured from isolated primary HLMCs cultured for 7 days in enriched media and incubated overnight with 300 ng/mL IgE. Cells were pre‐incubated for 5 min with Synta66 (10 *μ*mol/L) prior to stimulation with 3 *μ*g/mL anti‐IgE. Supernatants were harvested 24 h after challenge with anti‐IgE. A and C.) Mean mediator release in each condition from four lung donors. B and D.) Percentage inhibition of release in response to Anti‐IgE by Synta66, lines represents mean ±SEM. (E) Example results from one donor showing quantified cytokine levels displayed as integrated pixel density (1. CSFα, 2. MIF, 3. IL‐5, 4. IL‐6, 5. IL‐6, 6. Serpin E1.) (F) Percentage inhibition of cytokine release in response to Anti‐IgE by Synta66 from three lung donors, lines represent mean ±SEM. Results were analyzed, using one‐way ANOVA with Tukey posttest.

Prostaglandin D2 synthesis and secretion was measured from supernatants harvested 30 min after HLMCs were challenged with anti‐IgE. Synta66 had no significant effect on anti‐IgE stimulated PGD_2_ secretion in any of the donors tested (Fig. 7A–B)***.*** Confirmation of the dependency on calcium influx for PGD_2_ production was shown by performing the experiment in calcium‐free conditions; no PGD_2_ was detected (data not shown). Overall, these results indicate that Orai‐mediated calcium influx is not responsible for eicosanoid synthesis and release in HLMCs.

Following Fc*ε*RI stimulation for 24 h, mast cell supernatants were assessed for the release of inflammatory cytokines and chemokines using a human cytokine panel array (see methods). There was wide variation in cytokine release in response to anti‐IgE between 3 donors tested, and variation in the extent to which Synta66 inhibited it (Fig. 7)***.*** The maximum and most consistent inhibition by Synta66 was observed with MIF (macrophage migration inhibitory factor) secretion, with a mean percentage inhibition of 90%; CSF*α* (colony‐stimulating factor), IL‐5 and IL‐8 were all also inhibited by more than 60% and IL‐6 by 50%. For Serpin E1 and IL‐23, only ~20% inhibition was observed. To the best of our knowledge, this is the first demonstration that MIF, CSF*α*, Serpin E1, and IL‐23 are secreted from HLMCs following anti‐IgE activation, and that CSF*α* and MIF production are heavily dependent on Orai for synthesis and/or release.

In summary, the varying effects of Synta66 suggest differential regulation of cytokine and chemokine production via the range of signaling pathways initiated following Fc*ε*RI activation, each with differing amounts of involvement of Orai calcium signaling. Orai is not wholly and critically responsible for the anti‐IgE induced release of eicosanoids, cytokines and chemokines, but does have a critical role in the release of histamine and TNF‐*α*.

## Discussion

The results of this study show that Orai1, 2, 3; STIM1 and 2; TRPC 1 and 6 are expressed in LAD2 and HLMC cells. TRPC1 and 6 appear to have a predominantly intracellular localization and not to contribute significantly to antigen‐evoked calcium signaling following Fc*ε*RI receptor activation. Surface expression of TRPC1 channels is regulated through heteromultimerization with other TRPC channels or membrane trafficking. Heteromeric TRP channels containing TRPC1 are found at the plasma membrane, while homomeric TRPC1 channels are localized to intracellular compartments (Dietrich et al. 2014). Co‐expression with TRPC4 or TRPC5, but not TRPC6, leads to stable plasma membrane localization, coupling to G protein coupled receptors (GPCRs) and formation of receptors with reduced calcium permeability (Alfonso et al. 2008; Storch et al. 2012; Dietrich et al. 2014). Our evaluation of TRPC expression in HLMCs and LAD2 cells indicate that TRPC4 and TRPC5 are not present, which would be consistent with the lack of surface expression of TRPC1 observed by immunohistochemistry and support the notion that TRPC1 may form homomeric intracellular channels in HLMCs and LAD2 cells.

Intracellular localization of TRPC1 and TRPC6 channels has been observed previously in other cell types; controlling the trafficking of these channels to the surface and their interactions with proteins at the membrane thus plays a crucial role in regulating their cellular function (Ong et al. 2014; de Souza and Ambudkar 2014). Homomeric TRPC1 channels are confined to the endoplasmic reticulum and recycling endosomes and are therefore dependent on trafficking to the plasma membrane if they are to contribute to calcium influx (Alfonso et al. 2008; Dietrich et al. 2014; de Souza et al. 2015). Calcium influx through Orai and direct interactions between STIM and TRPC1 regulates the delivery of TRPC1 channels to specific domains within the plasma membrane (Ong et al. 2007; Zeng et al. 2008; Cheng et al. 2011). Spatial restriction of the channels within membrane microdomains impact upon their cellular function (Alicia et al. 2008). TRPC1 channels directed toward caveolin‐1‐enriched membrane domains and associated with the scaffolding protein homer (Pani et al. 2008, 2013) may allow preferential coupling to G protein‐coupled receptors (GPCRs) and PLCβ (Shi et al. 2016). Disrupting coupling of STIM1 to TRPC channels with STIM1‐KK684‐685EE is very effective in disrupting GPCR calcium signaling (Zeng et al. 2008; reviewed in Ong et al. 2016). Here, we show for the first time that antigen‐induced calcium influx following activation of FcεRI in human mast cells is completely unaffected by expression of STIM1‐KK^684‐685^EE indicating a lack of involvement of STIM1‐regulated TRPC1 downstream of receptor activation. FcεRI signaling is known to be spatially very restricted and organized through interactions with LAT, LAT2, and PLCγ1 (Gilfillan and Beaven 2011; Holowka and Baird 2015) and may therefore not provide the appropriate platform for recruiting TRPC1 channels to the membrane. Interestingly, expression of calveolin‐1 in rodent mast cells modifies calcium signals generated through a GPCR (Yeh et al. 2014) consistent with the idea that FcεRI and GPCRs may generate spatially and temporally distinct calcium signals to fine tune the responses of mast cells to distinct stimuli. Whether STIM‐regulated TRPC1 contribute to GPCR signaling in mast cells (Kuehn and Gilfillan 2007) remains to be determined.

TRPC6 was also found to be localized to an intracellular compartment in HLMCs and LAD2 cells. Consistent with this intracellular localization, application of a newly characterized TRPC3/6 selective agonist GSK‐2934A did not evoke calcium signals in mast cells although it was effective in TRPC6‐expressing HEK cells. Application of the TRPC6 antagonist GSK‐3503A to mast cells did not inhibit FcεRI‐initiated calcium signals. In agreement with our results, mast cells generated from TRPC6 knockout mice also show no alterations in FcεRI calcium signaling (Medic et al. 2013). Like with TRPC1, it has been reported that the surface expression and therefore the function of TRPC6 may be regulated through trafficking, GPCRs (Cayouette et al. 2004, 2010), and phosphoinositide 3‐kinase (Monet et al. 2012; Chaudhuri et al. 2016). A role for intracellular TRPC6 channels in innate immunity has also recently been described following their discovery on autophagosomes in alveolar macrophages (Riazanski et al. 2015); whether they similarly contribute to mast cell‐mediated host defense will require further investigation.

In agreement with previous published works by Ashmole et al. (2012, 2013), we found Orai channels to play a major role in FcεRI‐activated calcium influx and degranulation in HLMCs and LAD2 cells. Incubation of the cells with the Orai‐selective inhibitor Synta66 at 10 μmol/L inhibited the calcium signal measured in mast cells following antigen stimulation by approximately 70%; similar levels of inhibition were observed on secreted histamine and TNFα and some but not all cytokines. Owing to the limited solubility of Synta66, we could not explore whether higher concentrations would achieve a 100% block of either the calcium signals or mediator secretion, leaving the question of whether additional channels may be involved. As discussed above, all our evidence points against a role for TRPC channels in FcεRI‐regulated signaling. Moreover, patch clamp studies of currents evoked following store‐depletion in HLMCs by ourselves (data not shown) and (Ashmole et al. 2012, 2013) had all the biophysical characteristics of I_CRAC_, namely strong inward rectification, a reversal potential >40 mV, and high calcium selectivity (Prakriya and Lewis 2015), providing further evidence against a contribution of TRP channels to store‐operated calcium influx in human mast cells.

Taken together with the earlier study of Ashmole et al. 2012; our results show that FcεRI activated Orai‐mediated calcium influx directs the synthesis of LTC_4_, TNFα, IL‐5, IL‐6, IL‐8, Il‐13 CSFα, and MIF, but is not required for the synthesis of PGD_2_ and Serpin E1/PAI‐1 in HLMCs. Similar dependence on Orai signaling for antigen‐evoked LTC_4_, TNFα, and IL‐6 was reported for mast cells derived from Orai1 knockout mice (Vig et al. 2008). Secreted TNFα from mast cells may originate from a pre‐stored pool and thus would be expected to be inhibited by calcium influx through the same channels regulating degranulation and histamine i.e. Orai in human mast cells, as shown here. In late phase mast cell responses, however TNFα may be synthesized de novo and secreted along with other cytokines through an alternative pathway (Martin‐Avila et al. 2016), independent of calcium influx through Orai, such as shown here for Serpin E1. As with other de novo synthesized cytokines and chemokines, calcium‐sensitivity will be depend on the transcription factors driving their synthesis and integrated signaling with co‐stimulatory receptors (Gilfillan and Tkaczyk 2006; Klein et al. 2006; Pullen et al. 2012). In this regard, it is interesting to note that in bone marrow‐derived mast cells from TRPC1 knockout mice, an unexpected increase in antigen‐evoked CSF, TNFα and IL‐6 secretion, associated with increased activity of calcium‐regulated transcription factors, is observed which is enhanced by the presence of cKIT and dependent on IL‐3 (Medic et al. 2013). The enhanced and prolonged calcium influx observed in these cells is what would be expected by the loss of TRPC1 from heteromeric TRP channels (Dietrich et al. 2014). As discussed above, we did not find a role for TRPC in human mast cell signaling, thus whether the differences between our study and those of Medic et al. 2013 are due to the differences in the species, source and maturity of mast cells, all of which profoundly influence mast cell properties (Bischoff 2007) and/or presence of co‐stimulatory signals will require further investigation.

To summarize, the results presented in this study have shown important and novel evidence that Orai but not TRPC channels are contributing to Fc*ε*RI‐mediated calcium signaling in human mast cells. Taken together, these results have important consequences in highlighting the identity of potential therapeutic targets to treat mast cell‐mediated allergic disease, such as asthma. It can now be seen that selective compounds targeting Orai but not TRPC have the potential to be used as novel mast cell stabilizers/pre‐symptomatic asthma treatment.

## Conflict of Interests

Authors H.E.W and J.F received funding from GSK to support this work. H.E.W., M.B., K.A., C.G.S., J.P.M., and X.X.T are employees of GlaxoSmithKline.

## Supporting information




**Figure S1.** Immunocytochemistry showing TRPC1 expression in LAD2 cells. (A) anti‐TRPC1 staining, (B) anti‐IgG isotype control. Bar graph is quantification of mean staining intensity of cells in each conditions mean±SEM. Results were analyzed, using one‐way ANOVA with Bonferroni posttest. *= means were significantly different: **P < 0.01,***P < 0.001, ****P < 0.0001.

**Figure S2.** YFP‐tagged STIM1‐WT and STIM1 KK^684‐685^EE constructs translocate to PM following store depletion. Time lapse images of LAD2 cells transfected with STIM1 WT – YFP or STIM1 KK^684‐685^EE constructs. 2 *μ*mol/L Thapsigargin (TG) was applied to visualize translocation of STIM1 to the plasma membrane. Images were normalized for bleaching and are representative from 3 experiments *n* = 6. Results were analyzed, using an unpaired t‐test. **P < 0.01,***P < 0.001, ****P < 0.0001.

**Figure S3.** TRPC3/6 antagonist has no effect on P2Y or c‐kit receptor‐mediated calcium signaling. Calcium imaging of fura 2‐AM loaded HLMCs. (A) Mean calcium signal over time. 2 *μ*mol/L of GSK‐3503A antagonist was pre‐applied for 15 min before bath application of 100 *μ*mol/L ADP/100 ng/mL SCF, respectively, as indicated by the horizontal bars. (B) Scatter graph showing change in calcium signal (max‐basal) to ADP in each cell from all experiments, (C) 100 *μ*g/mL SCF applied as indicated by horizonal bars ‐ shows representative calcium signal traces and (**D)** shows a bar graph summarizing the normalized max and the area under the curve change in calcium signal to SCF. *n* > 50 for each condition over 3 or 4 HLMC donors. Results were analyzed, using student's unpaired t‐test/two‐way ANOVA with the Bonferroni posttest, as appropriate. ns*P* > 0.05

**Figure S4.** Synta66 has no effect on cell viability over a 24 h time period. Presence of the cell viability indicator enzyme LDH was quantified by colorimetric assay in primary isolated HLMCs following 24 h incubation with 3 *μ*g/mL anti‐IgE (black), 3 *μ*g/mL anti‐IgE + 10 *μ*mol/L Synta66 (red) or 10 *μ*mol/L Synta66 only, shown in comparison to the total enzyme from an equivalent number of lysed cells and spontaneous release. Each bar represents duplicate results from 10,000 mast cells.

**Figure S5.** GSK1702934A activated TRPC3 and TRPC6 channels heterologously expressed in HEK293 cells. (**A)** Time course of hTRPC3 activation by GSK1702934A at escalating concentrations. Top panel: Current amplitudes measured at ‐80 mV and +80 mV plotted against recording time. Bottom panel: Current/voltage relation of hTRPC3 in the absence and presence of 0.03, 0.1, and 0.3 *μ*mol/L GSK1702934A**. (B)** Time course of hTRPC6 activation by GSK1702934A at escalating concentrations (Top); current/voltage relation of hTRPC6 in the absence and presence of 0.1, 0.3, 1, and 3 *μ*mol/L GSK1702934A (Bottom). (**C)** GSK1702934A had no effect on the background current in HEK293 cells transduced with null BacMam virus (12%). (**D)** Concentration‐dependent response curve of GSK1702934A for hTRPC3 (*N* = 5) and hTRPC6 current (*N* = 7). (**E)** Concentration‐dependent response curve of GSK1702934A for rTRPC3 (*N* = 4) and rTRPC6 current (*N* = 5). (**F&G)** Compound structures for GSK1702934A and GSK2833503A, respectively.
