# Supplementary figures and images for: Orai and TRPC channel characterization in FcεRI‐mediated calcium signaling and mediator secretion in human mast cells
Source: Physiol Rep. 2017 Mar 14;5(5):e13166. doi: 10.14814/phy2.13166 (PMC5350174; doi:10.14814/phy2.13166)

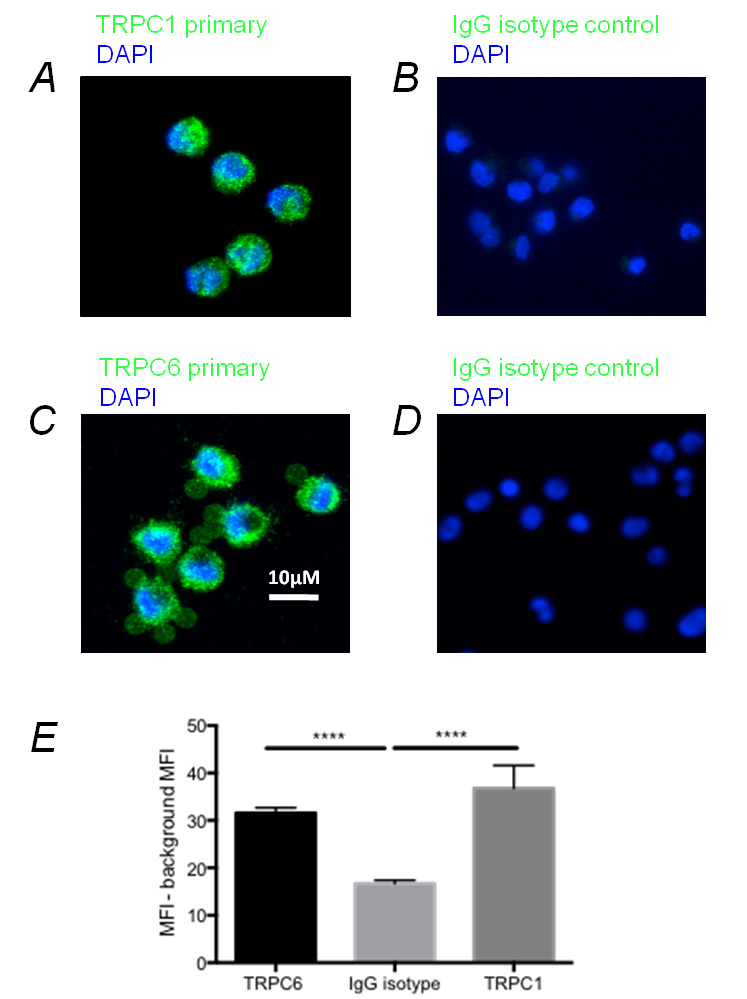

Supplement: Supplementary file 2 — Figure S2. YFP‐tagged STIM1‐WT and STIM1 KK684‐685EE constructs translocate to PM following store depletion. Time lapse images of LAD2 cells transfected with STIM1 WT – YFP or STIM1 KK684‐685EE constructs. 2 μmol/L Thapsigargin (TG) was applied to visualize translocation of STIM1 to the plasma membrane. Images were normalized for bleaching and are representative from 3 experiments n = 6. Results were analyzed, using an unpaired t‐test. **P < 0.01,***P < 0.001, ****P < 0.0001. [file PHY2-5-e13166-s002.tif]
